# A new computerized assessment battery for cognition (C-ABC) to detect mild cognitive impairment and dementia around 5 min

**DOI:** 10.1371/journal.pone.0243469

**Published:** 2020-12-11

**Authors:** Moeko Noguchi-Shinohara, Chiaki Domoto, Taketoshi Yoshida, Kozue Niwa, Sohshi Yuki-Nozaki, Miharu Samuraki-Yokohama, Kenji Sakai, Tsuyoshi Hamaguchi, Kenjiro Ono, Kazuo Iwasa, Ichiro Matsunari, Kiyonobu Komai, Hiroyuki Nakamura, Masahito Yamada

**Affiliations:** 1 Department of Neurology and Neurobiology of Aging, Kanazawa University Graduate School of Medical Sciences, Kanazawa University, Kanazawa, Japan; 2 Department of Preemptive Medicine for Dementia, Kanazawa University Graduate School of Medical Sciences, Kanazawa University, Kanazawa, Japan; 3 Department of Knowledge Science of Japan Advanced Institute of Science and Technology (JAIST), Nomi, Ishikawa, Japan; 4 Division of Neurology, Department of Internal Medicine, Showa University School of Medicine, Tokyo, Japan; 5 Department of Health and Medical Sciences, Ishikawa Prefectural Nursing University, Kahoku, Ishikawa, Japan; 6 Division of Nuclear Medicine, Department of Radiology, Saitama Medical University Hospital, Saitama, Japan; 7 Department of Neurology, Hokuriku Brain and Neuromuscular Disease Center, National Hospital Organization Iou National Hospital, Kanazawa, Japan; 8 Department of Public Health, Kanazawa University Graduate School of Advanced Preventive Medical Sciences, Kanazawa, Japan; 9 Kanazawa University Advanced Preventive Medical Sciences Research Center, Kanazawa, Japan; 10 Department of Environmental and Preventive Medicine, Kanazawa University Graduate School of Medical Sciences, Kanazawa University, Kanazawa, Japan; University of Padova, ITALY

## Abstract

This study aimed to develop a new computerized assessment battery for cognition (C-ABC) to detect mild cognitive impairment (MCI) and dementia. We performed C-ABC in subjects with dementia (*n* = 422), MCI (*n* = 145), and normal cognition (NC; *n* = 574), and analyzed by age stratum (50s, 60s, and 70–85 years). To distinguish MCI from NC, the C-ABC total combined score, which were calculated by dividing the C-ABC total score by the C-ABC required time, revealed the best area under the curves (AUC) at 0.838 and 0.735 in the 50s and 60s age groups, respectively; notably, this entire procedure took approximately 5 min. To distinguish dementia from NC and MCI, the partial items of C-ABC (items 3 + 6 combined score) revealed the best AUCs at 0.910, 0.874, and 0.882 in the 50s, 60s, and 70–85 age groups, respectively. Furthermore, the items 3 + 6 combined score established the best AUC at 0.794 in the 70–85 age group to distinguish MCI from NC; this entire procedure took around 2 min. Hence, this study suggests that C-ABC could be a useful tool for detecting dementia or MCI in a short time.

## Introduction

The number of patients with dementia has markedly increased as the population has aged around the world [1]. Thus, the importance of early detection of individuals with mild cognitive impairment (MCI) and dementia is now widely accepted.

The computerized cognitive test battery is superior over pencil-and-paper based classical cognitive test in terms of precision measurement of required time for cognitive tests and no need of trained medical staff [2]. Additionally, web-based testing provides the potential for large-scale screening of populations for cognitive function [3]. To detect MCI, the administration time for these computerized cognitive tests usually ranges from 10 to 30 minutes [4]. Although there is a lot of computerized cognitive test batteries, it was reported as difficult to select a test that would be most suitable for detecting dementia, and one that would be most suitable for detecting MCI, respectively [4]. Therefore, this study aimed to develop a sensitive and easily administered computerized cognitive test battery that could detect not only dementia but also MCI in a short time. In addition, we aimed to validate a new computerized assessment battery for cognition (C-ABC) to detect MCI and dementia with high sensitivity or specificity in older adults.

## Materials and methods

### Study population

We examined 758 consecutive patients who visited our memory clinic and underwent C-ABC (S1 Fig). Of these, we excluded 39 patients with psychiatric disorders (*n* = 36), such as depression, and disturbance of consciousness (*n* = 3). In addition, 18 patients were withdrawn from C-ABC because of hearing impairment (*n* = 9), difficulty of comprehension (*n* = 7), and visual impairment (*n* = 2). The remaining 701 patients were enrolled in this study, including patients with dementia (*n* = 422), MCI (*n* = 145), and normal cognition (NC; *n* = 134) (S1 Fig). The diagnoses of dementia and MCI were based on the guidelines of the Diagnostic and Statistical Manual of Mental Disorders, third edition, revised (DSM-III-R) [5] and the International Working Group on general criteria for MCI [6], respectively. The MCI criteria state that: 1) the individual should be judged as unhealthy using modalities other than those used to fulfill the DSM III-R dementia criteria; 2) the individual’s functional activities are mainly preserved or at least minimally impaired; and 3) the individual should have evidence of cognitive decline, either by self-assessment and/or by informant report in conjunction with deficits in objective cognitive tasks [6]. Among participants without dementia, a clinical dementia rating (CDR) [7] comprehensive score of 0.5 was used as the objective cognitive impairment value to denote cognitive and functional impairment consistent with MCI. Besides, we enrolled 440 individuals with NC from the Ishikawa Brain Imaging Study (S1 Fig) that was an imaging study aiming to develop imaging biomarkers for early and objective assessment of Alzheimer’s disease (AD) and other forms of neurodegenerative diseases using positron emission tomography and magnetic resonance imaging [8]. Of note, all subjects with NC, enrolled from our memory clinic and the Ishikawa Brain Imaging Study, were examined with the Mini-Mental State Examination (MMSE) [9] and CDR to assess cognitive profiles; MMSE cutoff point ≥ 24 of 30 and a CDR score of 0 suggested cognitively normal state [8, 9]. Additionally, subjects with NC had no history of psychiatric or neurological diseases. Furthermore, all subjects underwent medical screening, including a questionnaire about medical history and general and neurological examinations.

The age of the subjects ranged 23–95 years. As the cognitive function is susceptible to age, we analyzed the data by age stratum. We excluded patients aged <50 and >85 and analyzed only those aged 50–85 years, because only 1 patient with MCI aged <50 years and only 2 with NC aged >85 years. The remaining patients were divided into the following 3 age groups: 50s, 60s, and 70–85 years (70–85 group). Then, the dementia, MCI, and NC groups were created that matched age, education period, and gender by random sampling using SPSS software (version 23; SPSS Inc., Chicago, IL). After random sampling, there were 336 subjects with dementia, 137 with MCI, and 367 with NC (S1 Fig). Of 336 patients with dementia, 249 had AD, 23 had vascular dementia (VaD), 12 had mixed dementia (AD and VaD), 13 had dementia with Lewy bodies (DLB), 7 had frontotemporal dementia (FTD) and 32 had undetermined dementia. The diagnosis of AD was based on the criteria of the National Institute of Neurological and Communicative Disorders and stroke and the Alzheimer’s Disease and Related Disorders Association [10], VaD on the criteria of the National Institute of Neurological Disorders and Stroke and Association and Internationale pur la Rechrche et l’Enseignement en Neurosciences [11], DLB on the criteria of the third report of the DLB Consortium [12], and FTD on the diagnostic criteria of FTD [13].

This study was approved by the Medical Ethics Review Board of Kanazawa University (Kanazawa, Japan; approval number 1845). Written informed consent was obtained from all patients or their legal representatives.

### Computerized cognitive assessment battery

The C-ABC procedure was described to all subjects before the test administration. We usually examined C-ABC on a different day from MMSE, within a month. When the subjects were examined with C-ABC, a psychologist stayed to help them as necessary. All questions and instructions were presented in both a text on the PC screen (horizontal 80 cm × vertical 60 cm) and a verbal description through headphones (S2 Fig). All subjects were instructed to touch the touchscreen when they answer the C-ABC. The C-ABC comprised eight items, including sensorimotor skill, attention, orientation, immediate memory, and an arithmetic problem (Table 1).

**Table 1:** Items and contents of the computerized cognitive assessment battery.

Item	Task	Contents	Full score
1	Touching a moving target	The circle target was presented in different locations on the screen, one at a time, and the subject was asked to touch the circle target as quickly as possible.	10
2	The digits order	Nine digits (1–9) were presented at random positions on the screen and the subject was asked to touch the digits in sequential order as quickly as possible.	9
3	Time orientation	The subject was asked to choose the date of today (day, month, year, Japanese era name, and day of the week) from a list of candidates on the screen.	5
4	The letters-recognition memory test	Four Japanese letters of hiragana (“ri”, “na”, “ku”, and “me”) with meaningless relationships were presented on the screen for 5 seconds. Then, the subject was asked to select the recognized four letters from the Japanese syllabary table.	4
5	The numbers-recognition memory test	Three numbers without serial number were presented one by one on the screen. After 5 s, the subject was asked to select the three numbers in the correct order from the number plate.	3
6	The figures-recognition memory test	Four figures with different conditions in color and shape were presented on the screen. After 5 s, the subject was asked to select the recognized 4 figures from a set of 12 candidates.	4
7	The arithmetic problem	A shopping story was presented on the screen. The subject was asked about the total number of goods purchased from two stores. Then, the subject was asked to select the correct number from a set of four options.	1
8	Detecting a digit test	A table of random sequences of digits was presented on the screen. The subject was asked to detect and touch the digit (3) from the table. There were 4 items of digit 3 in the table.	4

The C-ABC total score ranges from 0 to 40 points. The time required for C-ABC (total required time; s) was automatically measured for each subject. The C-ABC total and each item combined scores were evaluated by dividing the C-ABC total and each item score by total and each item required time (s) and multiplying by 1000, respectively.

### Statistical analyses

First, we compared the clinical characteristics, MMSE score, C-ABC total score, each item score, required time, C-ABC total combined score, and each item combined score between NC, MCI, and dementia groups, using the Kruskal–Wallis test or χ^2^ test for each of the 50s, 60s and 70–85 groups. A correlation between C-ABC combined score and MMSE score was analyzed using Pearson’s correlation test.

Next, accuracies to diagnose dementia (dementia vs. NC and MCI) and MCI (MCI vs. NC) for the C-ABC total combined score, each item combined score, and MMSE were assessed by using the receiver-operating characteristic (ROC) analysis. Generally, a cognitive test that can distinguish dementia or MCI with high sensitivity and high specificity is the best. In this study, the optimal cutoff value (OCV) was defined as the cutoff point with the maximum value of the sum of the value of sensitivity and specificity. In addition, the ROC analyses revealed that items 3 (time orientation) and 6 (the figures-recognition memory test) were the best parameters among the item combined scores. We also performed the multivariate logistic regression analysis to evaluate which item combined score had large effect to distinguish dementia or MCI. We made the items 3 + 6 combined score which was calculated by dividing sum of items 3 and 6 scores by sum of items 3 and 6 required time and multiplying by 1000. The ROC analyses of the C-ABC total, each item, and items 3 + 6 combined scores revealed no ideal cutoff score with which both sensitivity and specificity were ≥0.9 in any age group.

Then, to distinguish MCI from NC and dementia from NC and MCI with high sensitivity, we defined the minimum score with sensitivity ≥0.9 as “sensitivity ≥0.9”. When C-ABC is performed as a screening test at hospitals followed by cognitive tests by trained medical staff, the cutoff score with high sensitivity should be used to decrease false negatives as much as possible.

Regarding specificities, we defined the maximum score with specificity ≥0.9 as “specificity ≥0.9” to distinguish MCI from NC and dementia from NC and MCI with high specificity. Older adults can perform C-ABC at home or any public facility other than hospitals because trained medical staffs are not needed. If older adults do self-inspection to detect dementia or MCI without following cognitive tests performed by trained medical staff, false positives seem to increase mental, time, and financial burdens. It must be important to set a high specificity cutoff value to decrease false positives.

In addition, we performed ten-fold cross-validation of ROC for the C-ABC combined score, by using all age groups data.

All data are presented as mean (SD) unless otherwise specified. We considered *P* < 0.05 as statistically significant. The ROC analyzes were performed with EZR (Saitama Medical Center, Jichi Medical University, Saitama, Japan), which is a graphical user interface for R (The R Foundation for Statistical Computing, Vienna, Austria). More precisely, it is a modified version of R commander designed to add statistical functions frequently used in biostatistics [14]. Other statistical analyses and random sampling were performed using the SPSS software package (version23; SPSS Inc., Chicago, IL).

## Results

### Subjects’ characteristics

The demographic and clinical characteristics of subjects are shown in Table 2.

**Table 2:** Clinical characteristics of subjects with NC, MCI, and dementia in the 50s, 60s, and 70–85 groups.

	NC	MCI	Dementia	*P* value
50s group				
n (women)	102 (57)	9 (1)	26 (12)	0.031
Age, mean (SD), years	55.40 (2.60)	56.56 (2.18)	56.31 (2.83)	0.154
Education, mean (SD), years	13.52 (2.44)	15.22 (2.77)	12.84 (2.30)	0.076
MMSE, mean (SD), points	29.05 (1.44)	27.67 (1.65)^a^	22.32 (4.75)^b^	< 0.001
60s group				
n (women)	165 (80)	34 (17)	77 (40)	0.881
Age, mean (SD), years	64.47 (2.81)	65.03 (3.08)	65.27 (2.54)	0.102
Education, mean (SD), years	12.65 (2.32)	13.76 (2.90)	12.41 (2.68)	0.069
MMSE, mean (SD), points	29.00 (1.31)	26.65 (2.18)^b^	20.45 (5.10)^b^^,^^c^	< 0.001
70–85 group				
n (women)	100 (64)	94 (46)	233 (119)	0.059
Age, mean (SD), years	74.99 (3.25)	75.78 (4.08)	76.04 (3.55)	0.052
Education, mean (SD), years	11.85 (2.49)	11.74 (2.42)	11.40 (2.68)	0.185
MMSE, mean (SD), points	28.17 (1.62)	25.73 (2.44)^b^	21.25 (8.48)^b^^,^^c^	< 0.001

*P* values determined by the Kruskal–Wallis test or *χ*^2^ test.

^a^*P* < 0.01 (compared with NC).

^b^*P* < 0.001 (compared with NC).

^c ^*P* < 0.001 (compared with MCI).

MCI, mild cognitive impairment; MMSE, Mini-Mental State Examination; NC, normal cognition

We observed no significant differences in gender ratio, mean age, and education period between dementia, MCI, and NC in the 60s and 70–85 groups. In the 50s group, the proportion of females was significantly lower in the MCI group than that in the dementia and NC groups.

### C-ABC total combined score

**Fig 1:**
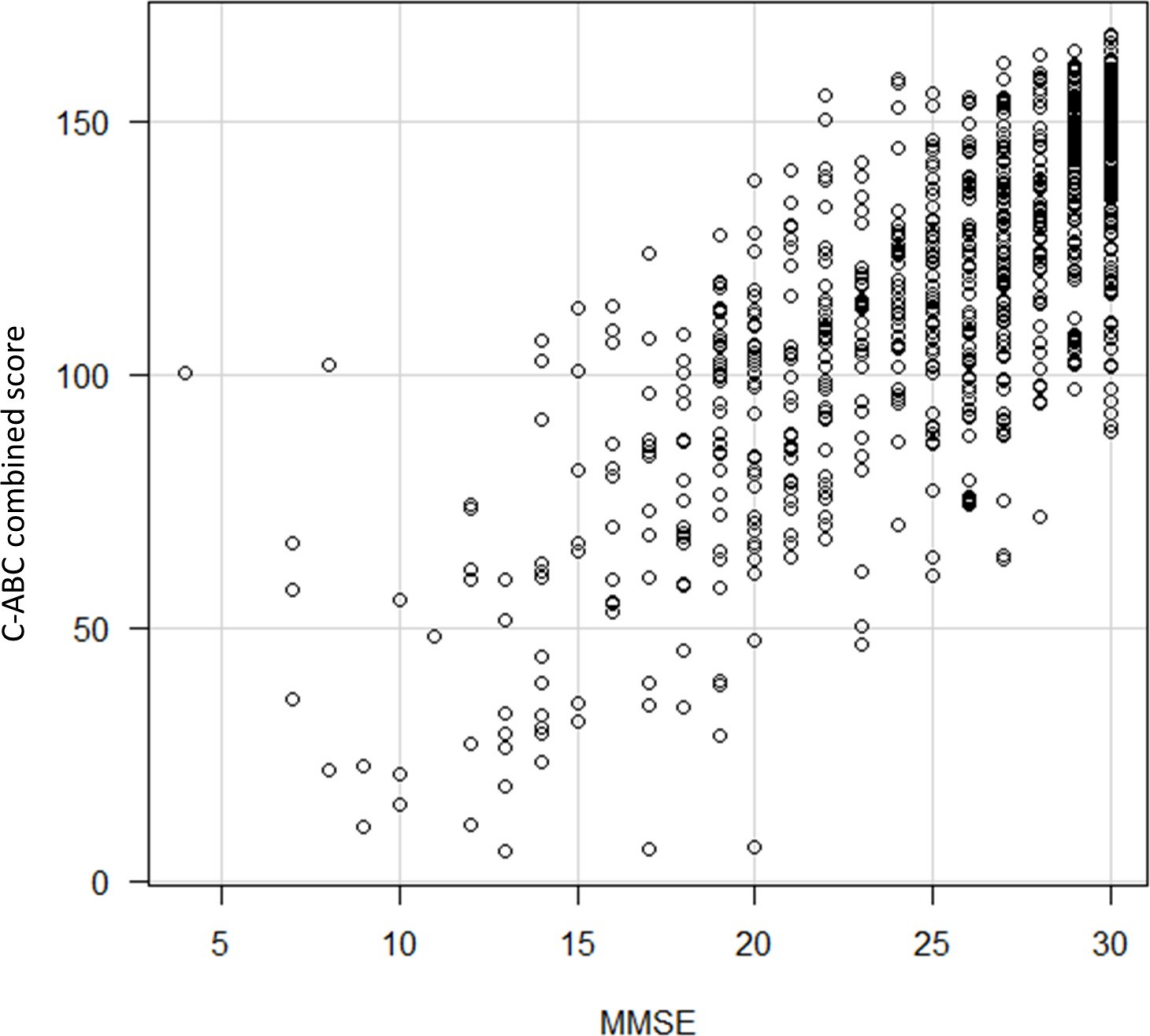
The correlation between C-ABC combined score and MMSE. C-ABC, computerized assessment battery for cognition; MMSE, Mini-Mental State Examination.

In all age groups, the mean C-ABC required time was approximately 5 min for dementia, MCI, and NC. The C-ABC combined score significantly correlated with the MMSE score (*r* = 0.753, *P* < 0.001; Fig 1).

**Fig 2:**
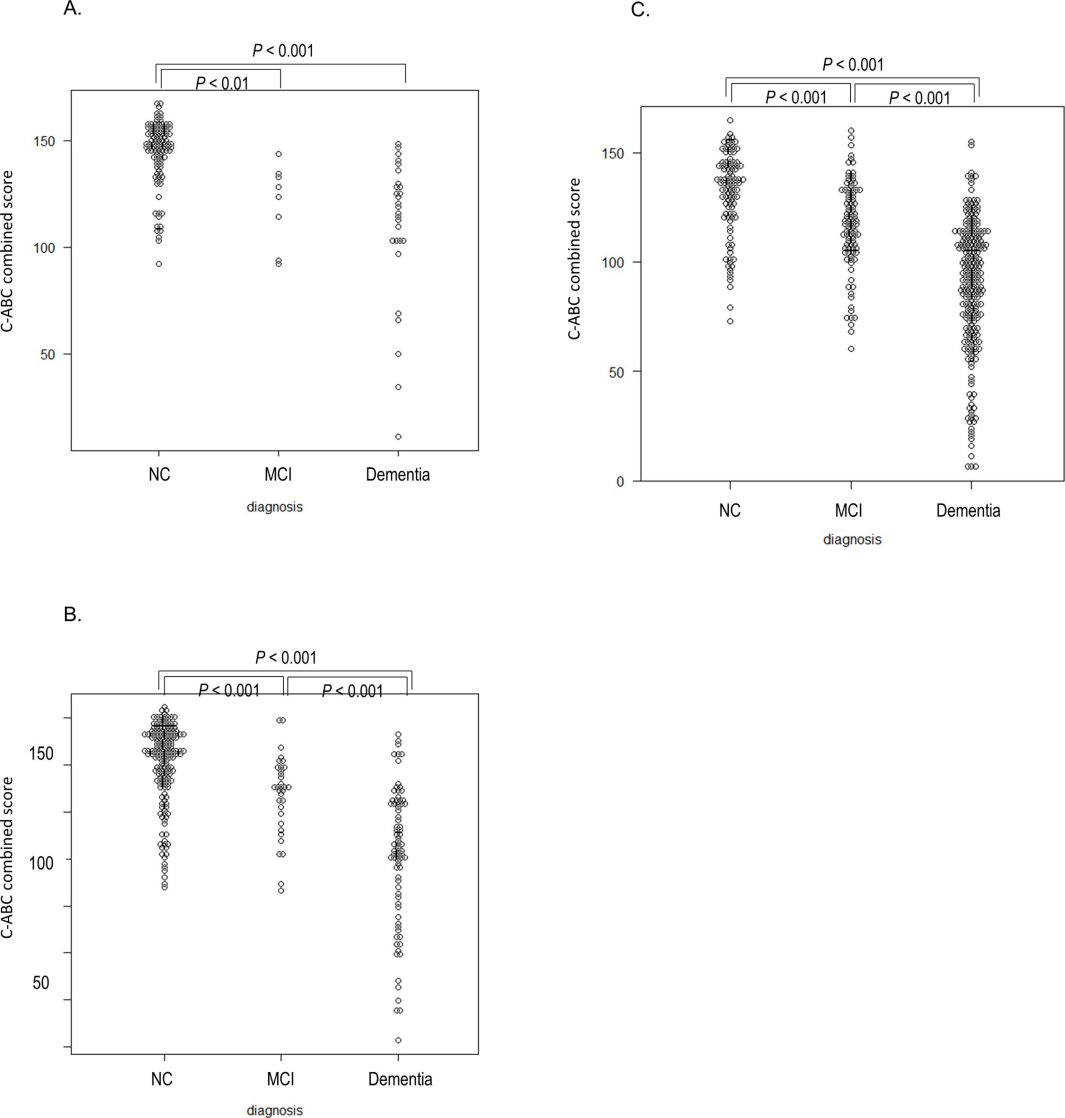
The score distribution of the C-ABC combined score in the 50s group (A), 60s group (B), and 70–85 group (C). The score distribution of C-ABC of NC, MCI, and dementia. C-ABC, computerized assessment battery for cognition; MCI, mild cognitive impairment; NC, normal cognition.

In addition, the C-ABC combined score was significantly lower in MCI and dementia than in NC in all age groups (Fig 2A–2C and S1 Table).

Furthermore, the C-ABC combined score was significantly lower in dementia than in MCI in the 60s and 70–85 groups (Fig 2B and 2C and S1 Table).

**Fig 3:**
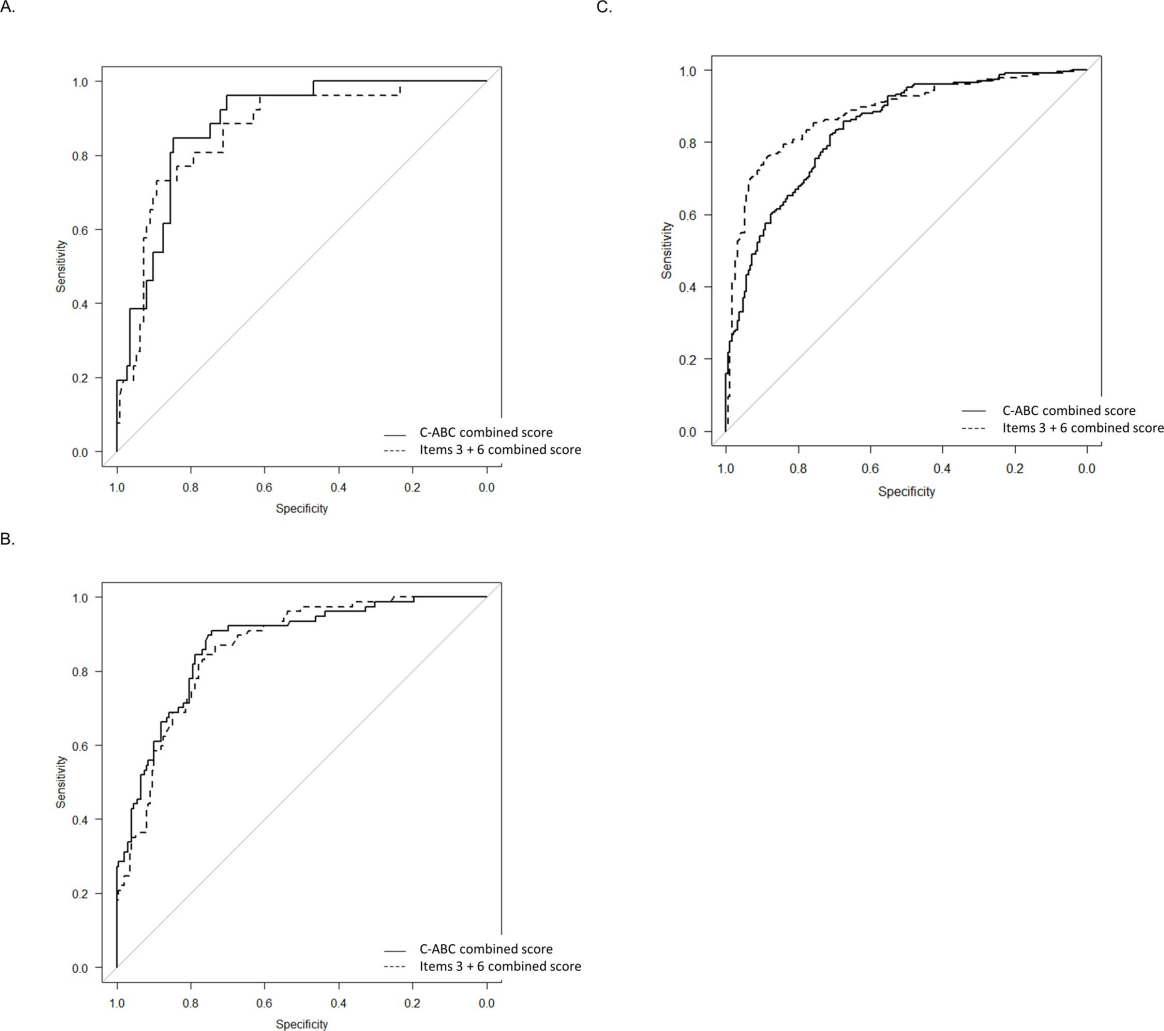
The ROC curves to distinguish dementia from MCI and NC in the 50s group (A), 60s group (B), and 70–85 group (C). Straight line, the C-ABC combined score; dotted line, the Item 3 + 6 combined score. C-ABC, computerized assessment battery for cognition; MCI, mild cognitive impairment; NC, normal cognition; ROC, receiver-operating characteristic.

When the C-ABC combined score was used, there was no ideal cutoff point with which both sensitivity and specificity were ≥0.9. The sensitivities/specificities at the OCV of the C-ABC combined score to distinguish dementia from MCI and NC were 0.84/0.84, 0.90/0.74, and 0.85/0.67 for the 50s, 60s, and 70–85 groups, respectively (Fig 3A–3C and Table 3).

**Table 3:** Measure of diagnostic accuracy of each item combined score, C-ABC total combined score, and item 3 + 6 combined score to distinguish dementia from MCI and NC and MCI from NC by ROC analyses.

	Dementia from MCI and NC				MCI from NC			
	AUC	95% CI	OCV	Sensitivity/ Specificity	AUC	95% CI	OCV	Sensitivity/ Specificity
50s group								
Item 1 combined score	0.660	0.537–0.784	256.41	0.38/ 0.91	0.687	0.537–0.838	290.69	0.77/ 0.61
Item 2 combined score	0.672	0.551–0.793	514.28	0.46/ 0.87	0.717	0.542–0.892	532.54	0.55/ 0.85
Item 3 combined score	0.888	0.816–0.960	104.71	0.88/ 0.80	0.748	0.613–0.884	117.64	0.77/ 0.77
Item 4 combined score	0.698	0.585–0.811	151.51	0.52/ 0.80	0.765	0.608–0.923	141.34	0.88/ 0.70
Item 5 combined score	0.579	0.437–0.720	112.36	0.57/ 0.63	0.583	0.388–0.778	111.94	0.55/ 0.69
Item 6 combined score	0.861	0.785–0.936	48.30	0.96/ 0.64	0.827	0.726–0.928	52.70	1.00/ 0.61
Item 7 combined score	0.607	0.464–0.749	40.00	0.46/ 0.85	0.736	0.539–0.932	38.31	0.55/ 0.90
Item 8 combined score	0.769	0.674–0.863	161.94	0.88/ 0.64	0.698	0.463–0.933	106.10	0.66/ 0.89
Total combined score	0.884	0.823–0.944	129.12	0.84/ 0.84	0.838	0.728–0.948	134.75	0.77/ 0.81
(Men and Women)								
Total combined score (Men)	0.799	0.680–0.918	128.68	0.85/ 0.75	0.761	0.601–0.922	143.47	0.87/ 0.60
Total combined score (Women)	0.957	0.912–1.000	138.78	1.00/ 0.82	0.947	–*	128.83	1.00/ 0.94
Item 3 + 6 combined score	0.910	0.858–0.963	60.92	0.92/ 0.78	0.814	0.724–0.904	71.42	1.00/ 0.67
60s group								
Item 1 combined score	0.705	0.636–0.774	280.11	0.70/ 0.61	0.614	0.511–0.716	275.48	0.55/ 0.69
Item 2 combined score	0.756	0.688–0.824	489.13	0.55/ 0.86	0.640	0.541–0.740	588.23	0.73/ 0.58
Item 3 combined score	0.856	0.808–0.904	102.24	0.85/ 0.75	0.728	0.626–0.831	98.03	0.55/ 0.86
Item 4 combined score	0.796	0.737–0.856	129.31	0.76/ 0.70	0.619	0.518–0.720	148.14	0.73/ 0.51
Item 5 combined score	0.659	0.581–0.736	105.63	0.40/ 0.89	0.570	0.449–0.691	110.29	0.50/ 0.70
Item 6 combined score	0.828	0.775–0.881	39.37	0.83/ 0.73	0.683	0.583–0.783	49.50	0.67/ 0.69
Item 7 combined score	0.703	0.624–0.782	32.78	0.55/ 0.88	0.506	0.386–0.627	44.64	0.12/ 0.99
Item 8 combined score	0.673	0.595–0.751	129.87	0.49/ 0.82	0.574	0.473–0.675	187.79	0.82/ 0.39
Total combined score	0.872	0.827–0.918	130.36	0.90/ 0.74	0.735	0.650–0.820	142.90	0.88/ 0.59
(Men and Women)								
Total combined score (Men)	0.868	0.805–0.932	125.57	0.89/ 0.76	0.623	0.477–0.768	144.28	0.76/ 0.47
Total combined score (Women)	0.879	0.815–0.944	129.92	0.90/ 0.78	0.852	0.777–0.927	142.90	1.00/ 0.69
Item 3 + 6 combined score	0.874	0.833–0.915	59.34	0.87/ 0.76	0.735	0.643–0.827	67.56	0.70/ 0.71
70–85 group								
Item 1 combined score	0.681	0.630–0.732	271.73	0.79/ 0.51	0.579	0.498–0.661	249.37	0.42/ 0.77
Item 2 combined score	0.703	0.653–0.752	523.25	0.78/ 0.53	0.606	0.526–0.686	529.41	0.60/ 0.60
Item 3 combined score	0.879	0.847–0.912	86.58	0.82/ 0.79	0.738	0.668–0.807	93.16	0.47/ 0.90
Item 4 combined score	0.699	0.649–0.748	114.06	0.68/ 0.63	0.603	0.523–0.682	118.34	0.52/ 0.67
Item 5 combined score	0.674	0.623–0.724	109.89	0.67/ 0.59	0.488	0.406–0.570	75.75	0.12/ 0.93
Item 6 combined score	0.760	0.715–0.806	26.24	0.66/ 0.74	0.732	0.663–0.802	50.82	0.90/ 0.46
Item 7 combined score	0.645	0.594–0.696	36.23	0.71/ 0.57	0.609	0.531–0.687	36.76	0.55/ 0.66
Item 8 combined score	0.658	0.607–0.709	85.28	0.32/ 0.92	0.544	0.463–0.626	161.29	0.60/ 0.53
Total combined score	0.840	0.803–0.877	117.44	0.85/ 0.67	0.722	0.650–0.795	124.91	0.66/ 0.72
(Men and Women)								
Total combined score (Men)	0.821	0.764–0.879	114.30	0.79/ 0.71	0.694	0.574–0.813	128.25	0.77/ 0.64
Total combined score (Women)	0.853	0.804–0.902	117.43	0.90/ 0.67	0.745	0.653–0.838	136.46	0.87/ 0.56
Item 3 + 6 combined score	0.882	0.850–0.915	40.91	0.75/ 0.89	0.794	0.731–0.857	62.11	0.78/ 0.69
All age groups								
Total combined score	0.875	0.852–0.898	128.47	0.92/ 0.67	0.782	0.739–0.825	136.47	0.81/ 0.67
(Men and Women)								
50–64 group								
Total combined score	0.886	0.839–0.933	129.59	0.86/ 0.83	0.787	0.700–0.874	143.47	0.87/ 0.68
(Men and Women)								
65–85 group								
Total combined score	0.854	0.824–0.884	117.44	0.83/ 0.72	0.741	0.684–0.798	132.47	0.77/ 0.64
(Men and Women)								

AUC, area under the curve; C-ABC, computerized assessment battery for cognition; MCI, mild cognitive impairment; NC, normal cognition; OCV, optimal cutoff value (in this study, OCV implies the value in which the sum of sensitivity and specificity reaches the maximum); ROC, receiver-operating characteristic analysis

* We could not calculate because number of MCI patients was 1.

**Fig 4:**
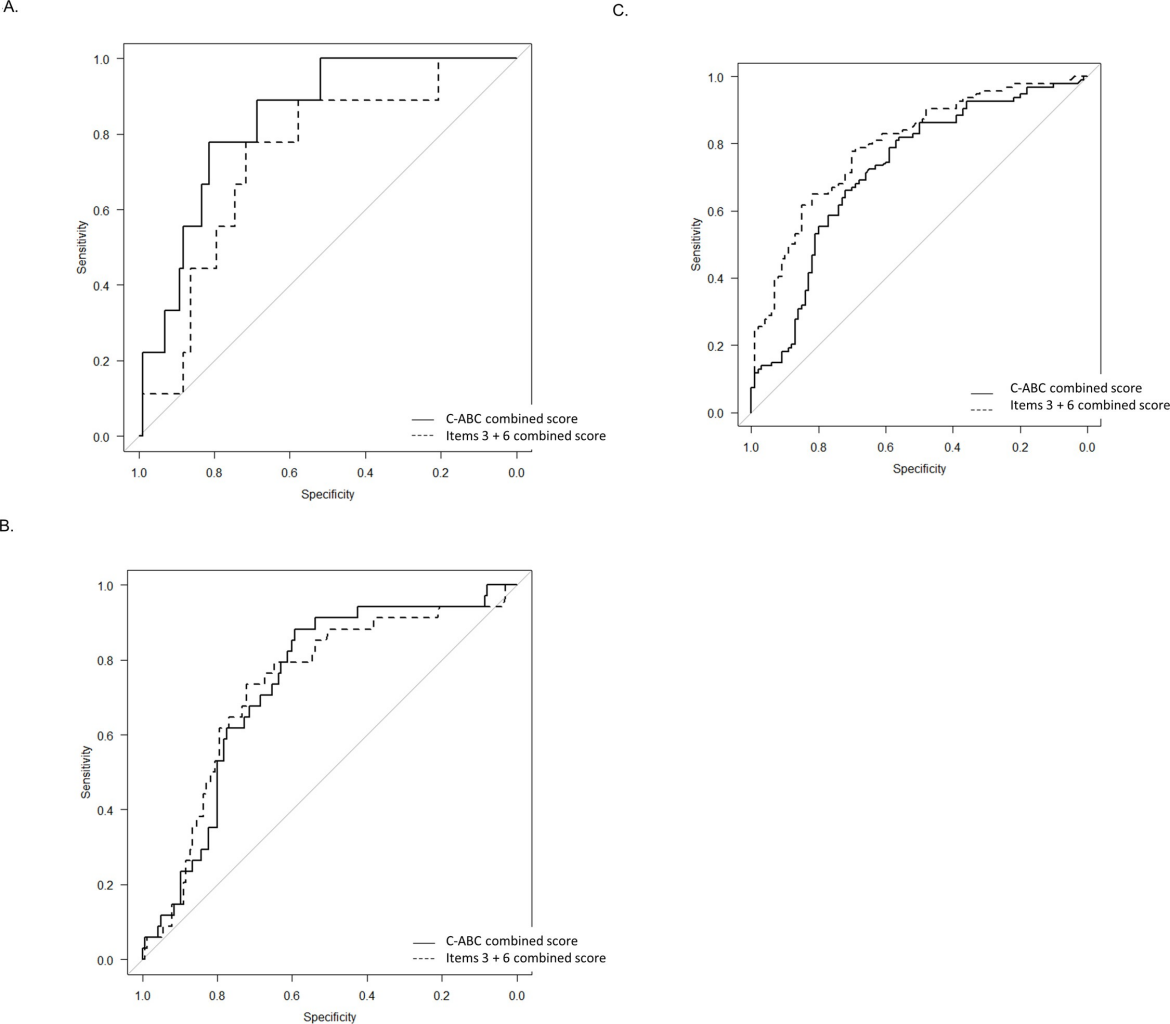
The ROC curves to distinguish MCI from NC in the 50s group (A), 60s group (B) and 70–85 group (C). Straight line, the C-ABC combined score; dotted line, the Item 3 + 6 combined score. C-ABC, computerized assessment battery for cognition; MCI, mild cognitive impairment; NC, normal cognition; ROC, receiver-operating characteristic.

For distinguishing MCI from NC, the sensitivities/specificities at the OCV were 0.77/0.81, 0.88/0.59, and 0.66/0.72 for the 50s, 60s, and 70–85 groups, respectively (Fig 4A–4C and Table 3).

Additionally, we performed ten-fold cross-validation of ROC for the C-ABC combined score using all age groups. The mean sensitivities/ specificities at the OCV of the C-ABC combined score to distinguish dementia from MCI and NC and MCI from NC were 0.91/ 0.63 and 0.80/0.59, respectively.

### Each item score and items 3 + 6 combined score

Among items, the mean score and percentage of full score of item 3 (time orientation) were very low in dementia than that in NC and MCI (S1 Table). As item 6 (the figures-recognition memory test) was relatively difficult, only 72.5% of NC subjects in the 50s group could answer all correctly (S1 Table). Then, the mean score and percentage of full score of item 6 were very low in MCI and dementia than that in NC in all age groups, and the required time was prolonged, especially in the 50s group. Almost all item combined scores were significantly lower in dementia than that in NC in all age groups and were significantly lower in dementia than that in MCI in the 60s and 70–85 groups (S2 Table). Among items, only item 6 combined score was significantly lower in MCI than in that NC in all age groups (S2 Table). Multivariate logistic regression analyses showed that item 3 combined score had the most effect for distinguishing dementia, and item 6 combined score had the second most effect (S3 Table). Additionally, for distinguishing MCI, both of item 3 and 6 combined scores had almost equally the most effect in the multivariate logistic regression analysis (S2 Table). The diagnostic accuracy of each item combined score is shown in Table 3. In this study, we could not find the ideal cutoff point with which both sensitivity and specificity were ≥0.9 among item combined scores (Table 3). Regarding the area under the curve (AUC) using the score of each item combined score for C-ABC, item 3 combined score exhibited the maximum AUC to distinguish dementia from MCI and NC in all age groups, and MCI from NC in the 60s and 70–85 groups (Table 3). Besides, item 6 combined score was the best parameter with the maximum AUC to distinguish MCI from NC in the 50s group (Table 3). Therefore, we made the items 3 + 6 combined score. The mean required time to perform items 3 and 6 was approximately 2 min for dementia, MCI, and NC in each age group. Moreover, the items 3 + 6 combined score significantly correlated with the MMSE score (*r* = 0.740, *P* < 0.0001; S3 Fig). In addition, the items 3 + 6 combined score was significantly lower in MCI and dementia than that in NC in all age groups (S4A–S4C Fig and S1 Table). Besides, the items 3 + 6 combined score was significantly lower in dementia than that in MCI in the 60s and 70–85 groups (S4B and S4C Fig and S1 Table). When the items 3 + 6 combined score was used, no ideal cutoff point was found with which both sensitivity and specificity were ≥ 0.9. Comparison of the diagnostic accuracies between C-ABC total combined score and items 3 + 6 combined score revealed that the items 3 + 6 combined score exhibited a marginally better AUC to distinguish dementia from MCI and NC in all age groups (Fig 3A–3C and Table 3), and the items 3 + 6 combined score was better for distinguishing MCI from NC in the 70–85 group. In the 50s and 60s groups, however, the C-ABC total combined score was better to distinguish MCI from NC (Fig 3A–3C and Table 3).

### Detection of MCI/ dementia with sensitivity ≥0.9

We analyzed the cutoff value for sensitivity ≥0.9. When the C-ABC total combined score was used and the cutoff points of sensitivity ≥0.9 were set to 138, 132, and 123 for the 50s, 60s, and 70–85 groups, respectively, the sensitivities to distinguish dementia from MCI and NC were 0.91, 0.90, and 0.90, respectively (Table 4).

**Table 4:** Measure of diagnostic accuracy of C-ABC combined score and MMSE for separation of MCI from NC and dementia from MCI and NC by ROC analyses.

	MCI from NC			Dementia from MCI and NC		
C-ABC combined score	Specificity ≥ 0.9	OCV	Sensitivity ≥ 0.9	Specificity ≥ 0.9	OCV	Sensitivity ≥ 0.9
50s group						
cutoff point	114	134	147	111	129	138
Sensitivity	0.33	0.77	1.00	0.46	0.84	0.92
Specificity	0.93	0.81	0.51	0.91	0.84	0.72
60s group						
cutoff point	110	142	146	107	130	132
Sensitivity	0.15	0.88	0.91	0.55	0.90	0.90
Specificity	0.90	0.59	0.46	0.90	0.74	0.69
70–85 group						
cutoff point	101	124	138	96	117	123
Sensitivity	0.18	0.66	0.90	0.54	0.85	0.90
Specificity	0.91	0.72	0.36	0.90	0.68	0.56
	MCI from NC			Dementia from MCI and NC		
MMSE	Specificity ≥ 0.9	OCV	Sensitivity ≥ 0.9	Specificity ≥ 0.9	OCV	Sensitivity ≥ 0.9
50s group						
cutoff point	26	28	30	26	28	29
Sensitivity	0.22	0.77	1.00	0.68	1.00	1.00
Specificity	0.91	0.78	0.00	0.90	0.74	0.53
60s group						
cutoff point	26	28	30	25	26	27
Sensitivity	0.47	0.79	1.00	0.77	0.89	0.97
Specificity	0.93	0.75	0.00	0.91	0.86	0.77
70–85 group						
cutoff point	25	27	29	22	24	26
Sensitivity	0.44	0.72	0.99	0.65	0.81	0.93
Specificity	0.92	0.69	0.24	0.93	0.87	0.65

C-ABC: Computerized Assessment Battery for Cognition; MCI: mild cognitive impairment; MMSE: Mini-Mental State Examination; NC: normal cognition; OCV: optimal cutoff value. In this study, OCV means the value, in which sum of sensitivity and specificity reaches the maximum; ROC: receiver operating characteristic analysis.

Likewise, when the cutoff points of sensitivity ≥0.9 to distinguish MCI for NC were set to 147, 146, and 138 for the 50s, 60s, and 70–85 groups, respectively, the sensitivities were 1.00, 0.91, and 0.90, respectively (Table 4). In addition, when using the MMSE score similarly, the cutoff points of sensitivity ≥0.9 were 30, 30, and 29 for the 50s, 60s, and 70–85 groups, respectively, and the sensitivities for detecting MCI were 1.00, 1.00, and 0.99, respectively (Table 4).

When the items 3 + 6 combined score was used, the cutoff points of sensitivity ≥0.9 were set to 68.23, 65.62, and 57.85 for the 50s, 60s, and 70–85 groups, respectively, and the sensitivities to distinguish dementia from MCI and NC were 0.92, 0.90, and 0.90, respectively (S2 Table). Furthermore, when the cutoff points of sensitivity ≥0.9 to distinguish MCI for NC were set to 80.86, 80.07 and 69.74, for the 50s, 60s and 70–85 groups, respectively, the sensitivities were 1.00, 0.91 and 0.90 respectively (S2 Table).

### Detection of MCI/ dementia with specificity ≥0.9

We also analyzed the cutoff value for specificity ≥0.9. When the C-ABC combined score was used and the cutoff points of specificity ≥0.9 were set to 111, 107, and 96 for the 50s, 60s, and 70–85 groups, respectively, the specificities to distinguish dementia from MCI and NC were 0.91, 0.90, and 0.90, respectively (Table 4). Similarly, when the cutoff points of specificity ≥0.9 to distinguish MCI for NC were set to 114, 110, and 101, for the 50s, 60s, and 70–85 groups, respectively, the specificities were 0.93, 0.90, and 0.91, respectively (Table 4).

When the items 3 + 6 combined score was used, and the cutoff points of specificity ≥0.9 were set to 48.82, 36.17, and 36.04 for the 50s, 60s, and 70–85 groups, respectively, the specificities to distinguish dementia from MCI and NC were 0.90, 0.90, and 0.93, respectively (S2 Table).

## Discussion

The major findings of this study were as follows: (i) the C-ABC only took approximately 5 min; (ii) the C-ABC total combined score cutoff points with high sensitivities or specificities could be set according to the purpose of usage; and (iii) the items 3 + 6 combined score, which took only 2 min, displayed better diagnostic accuracies to detect dementia compared with the C-ABC combined score.

Regarding comparison with MMSE, the C-ABC combined score and the items 3 + 6 combined score highly correlated with MMSE scores. Some cognitive domains of C-ABC, such as attention, orientation, and recognition memory, are similar to MMSE. Regarding sensitivities, setting the cutoff value of 30 was essential to attain the sensitivity of ≥0.9 in MMSE. As full score of MMSE is 30 points, a cutoff of 30 points is irrelevant to taking the test, and detecting MCI by MMSE with high sensitivity would be difficult. It was reported that MMSE may not detect MCI [15] or mild dementia [16].

The C-ABC total combined score using a high sensitivity cutoff value ≥0.9 is appropriate for screening tests, followed by cognitive tests by trained medical staff. If C-ABC is performed outside the hospital or health examination in the absence of following cognitive tests, it is essential to set a high specificity cutoff value to decrease false positives. In such cases, false negatives would increase; however, repeated C-ABC probably will be able to detect MCI and dementia early.

In addition, C-ABC can be completed within a short time (approximately 5 min); the administration time of C-ABC was shorter than that of MMSE (approximately 10 min for older person [15]). In addition, the AUC of the item 3 + 6 combined score in our study was higher than that of the C-ABC combined score to detect dementia in all age groups. Among items, the mean score and percentage of full score of item 3 (time orientation) were very low in dementia, and those of item 6 (the figures-recognition memory test) were very low in MCI and dementia. Although patients with dementia usually have disorientation and severe memory disturbance, those with MCI and NC rarely have both. Thus, item 3 that asks time orientation and item 6 that requires to memory 4 figures in detail would be beneficial in distinguishing dementia from MCI and NC. In our study, items 3 + 6 were performed in only around 2 min. With regard to computerized cognitive battery, the Cogstate Brief Battery, which comprises four cognitive tasks and was reported to be able to distinguish patients with MCI from healthy older adults requires 10 min [17]. A brief computerized paired-associate test (the Miami Test of Semantic Interference and Learning) to detect MCI requires around 9 min [18]. As computerized cognitive test batteries, such as C-ABC, could be performed on numerous people simultaneously in a short time, it is beneficial for screening both MCI and dementia.

This study has some limitations. First, the sample size was relatively small. Thus, further longitudinal studies examining a larger sample size with various cognitive impairments are needed to measure the reproducibility and broad application of C-ABC. Second, the items 3 + 6 combined score displayed better accuracies to distinguish dementia compared with the C-ABC combined score. However, we did not examine measurements using only items 3 and 6. Thus, additional studies using only items 3 and 6 are warranted to elucidate the utility of items 3 and 6.

In conclusion, we developed the new C-ABC that could detect patients with MCI and dementia in older adults with high sensitivities or specificities in approximately 5 min. Furthermore, the items 3 + 6 combined score could detect patients with dementia in only around 2 min.

## Supporting information

S1 Fig The flowchart of the subjects. We excluded subjects aged <50 and >85, and analyzed only those 50–85 years, because only 1 subject with MCI aged <50 years and only 2 with NC aged >85 years. We excluded 194 subjects aged <50 years and 2 aged >85 years from the NC group (*1). We excluded 1 subject aged <50 years and 4 aged >85 years from the MCI group (*2). We excluded 6 subjects aged <50 years and 29 aged >85 years from the dementia group (*3). In addition, the dementia, MCI, and NC groups were created that matched age, education period, and gender by random sampling using SPSS software (version 23; SPSS Inc., Chicago, IL). Consequently, we excluded 11, 3, and 51 subjects from the NC (*1), MCI (*2), and dementia (*3) groups, respectively. Overall, we excluded 207 subjects from the NC group, 8 from the MCI group, and 86 from the dementia group, and finally examined 367 subjects as the matched NC group, 137 as the matched MCI group, and 336 as the matched dementia group. C-ABC, computerized assessment battery for cognition; MCI, mild cognitive impairment; NC, normal cognition.
(TIF)

S2 Fig The computerized assessment battery for cognition. The figures-recognition memory test (item 6). On the PC screen, the question "Please touch the figure with the same color and shape, which you memorized earlier" has been presented.
(TIF)

S3 Fig The correlation between the items 3 + 6 combined score and MMSE. MMSE, Mini-Mental State Examination.
(TIF)

S4 Fig The score distribution of the items 3 + 6 combined score in the 50s group (A), 60s group (B), and 70–85 group (C). The score distribution of C-ABC of NC, MCI, and dementia. C-ABC, computerized assessment battery for cognition; MCI, mild cognitive impairment; NC, normal cognition.
(TIF)

S1 Table The mean score and percentage of full score, required time, combined score* for each item, all items, and items 3 + 6 in subjects with NC, MCI, and dementia for the 50s, 60s, and 70–85 groups. (DOCX)

S2 Table Multiple logit estimates for dementia or MCI. (DOCX)

S3 Table Measure of the diagnostic accuracy of items 3 + 6 combined score to distinguish MCI from NC and dementia from MCI and NC by ROC analyses. (DOCX)

S1 Dataset (XLSX)

## References

[1] Prince M, Bryce R, Albanese E, Wimo A, Ribeiro W, Ferri CP. The global prevalence of dementia: a systematic review and metaanalysis. Alzheimers Dement. 2013;9(1):63-75.e2. Epub 2013/01/12. 10.1016/j.jalz.2012.11.007 . 23305823

[2] Wild K, Howieson D, Webbe F, Seelye A, Kaye J. Status of computerized cognitive testing in aging: a systematic review. Alzheimers Dement. 2008;4(6):428–37. Epub 2008/11/18. 10.1016/j.jalz.2008.07.003 . 19012868 PMC2645803

[3] Feenstra HEM, Vermeulen IE, Murre JMJ, Schagen SB. Online cognition: factors facilitating reliable online neuropsychological test results. Clin Neuropsychol. 2017; 31 (1): 59–84. 10.1080/13854046.2016.1190405 27266677

[4] Zygouris S, Tsolaki M. Computerized cognitive testing for older adults: A review. Am J Alzheimers Dis Other Demen. 2015; 30 (1): 13–28. 10.1177/1533317514522852 24526761 PMC10852880

[5] American Psychiatric Association. Diagnostic and statistical manual of mental disorders, 3rd ed, revised. 1987; American Psychiatric Association, Washington, DC.

[6] Winblad B, Palmer K, Kivipelto M, Jelic V, Fratiglioni L, Wahlund LO, et al Mild cognitive impairment—beyond controversies, towards a consensus: report of the International Working Group on Mild Cognitive Impairment. J Intern Med. 2004;256(3):240–6. Epub 2004/08/25. 10.1111/j.1365-2796.2004.01380.x . 15324367

[7] Morris JC. The Clinical Dementia Rating (CDR): current version and scoring rules. Neurology. 1993;43(11):2412–4. Epub 1993/11/01. 10.1212/wnl.43.11.2412-a . 8232972

[8] Komatsu J, Matsunari I, Samuraki M, Shima K, Noguchi-Shinohara M, Sakai K, et al Optimization of DARTEL Settings for the Detection of Alzheimer Disease. Am J Neuroradiol. 2018;39(3):473–8. Epub 2018/02/09. 10.3174/ajnr.A5509 . 29419401 PMC7655334

[9] Folstein MF, Folstein SE, McHugh PR. "Mini-mental state". A practical method for grading the cognitive state of patients for the clinician. J Psychiatr Res. 1975;12(3):189–98. Epub 1975/11/01. 10.1016/0022-3956(75)90026-6 . 1202204

[10] McKhann G, Drachman D, Folstein M, Katzman R, Price D, Stadlan EM. Clinical diagnosis of Alzheimer's disease: report of the NINCDS-ADRDA Work Group under the auspices of Department of Health and Human Services Task Force on Alzheimer's Disease. Neurology. 1984;34(7):939–44. Epub 1984/07/01. 10.1212/wnl.34.7.939 . 6610841

[11] Roman GC, Tatemichi TK, Erkinjuntti T, Cummings JL, Masdeu JC, Garcia JH, et al Vascular dementia: diagnostic criteria for research studies. Report of the NINDS-AIREN International Workshop. Neurology. 1993;43(2):250–60. Epub 1993/02/01. 10.1212/wnl.43.2.250 . 8094895

[12] McKeith IG, Dickson DW, Lowe J, Emre M, O'Brien JT, Feldman H, et al Diagnosis and management of dementia with Lewy bodies: third report of the DLB Consortium. Neurology. 2005;65(12):1863–72. Epub 2005/10/21. 10.1212/01.wnl.0000187889.17253.b1 . 16237129

[13] Neary D, Snowden JS, Gustafson L, Passant U, Stuss D, Black S, et al Frontotemporal lobar degeneration: a consensus on clinical diagnostic criteria. Neurology. 1998;51(6):1546–54. Epub 1998/12/17. 10.1212/wnl.51.6.1546 . 9855500

[14] Kanba Y. Investigation of the freely available easy-to-use software ‘EZR’ for medical statistics. Bone Marrow Transplant. 2013;48:452–458. Epub 2012/12/03. 10.1038/bmt.2012.244 . 23208313 PMC3590441

[15] Loewenstein DA, Barker WW, Harwood DG, Luis C, Acevedo A, Rodriguez I, et al Utility of a modified Mini-Mental State Examination with extended delayed recall in screening for mild cognitive impairment and dementia among community dwelling elders. Int J Geriatr Psychiatry. 2000;15(5):434–40. Epub 2000/05/24. 10.1002/(sici)1099-1166(200005)15:5<434::aid-gps137>3.0.co;2-2 . 10822242

[16] Feher EP, Mahurin RK, Doody RS, Cooke N, Sims J, Pirozzolo FJ. Establishing the limits of the Mini-Mental State. Examination of 'subtests'. Arch Neurol. 1992;49(1):87–92. Epub 1992/01/01. 10.1001/archneur.1992.00530250091022 . 1728269

[17] Maruff P, Lim YY, Darby D, Ellis KA, Pietrzak RH, Snyder PJ, et al Clinical utility of the cogstate brief battery in identifying cognitive impairment in mild cognitive impairment and Alzheimer's disease. BMC Psychol. 2013;1(1):30 Epub 2013/01/01. 10.1186/2050-7283-1-30 . 25566378 PMC4269990

[18] Curiel RE, Crocco E, Rosado M, Duara R, Greig MT, Raffo A, et al A Brief Computerized Paired Associate Test for the Detection of Mild Cognitive Impairment in Community-Dwelling Older Adults. J Alzheimers Dis. 2016;54(2):793–9. Epub 2016/08/29. 10.3233/JAD-160370 . 27567839 PMC5610962

