# Supplementary figures and images for: A new computerized assessment battery for cognition (C-ABC) to detect mild cognitive impairment and dementia around 5 min
Source: PLoS One. 2020 Dec 11;15(12):e0243469. doi: 10.1371/journal.pone.0243469 (PMC7732101; doi:10.1371/journal.pone.0243469)

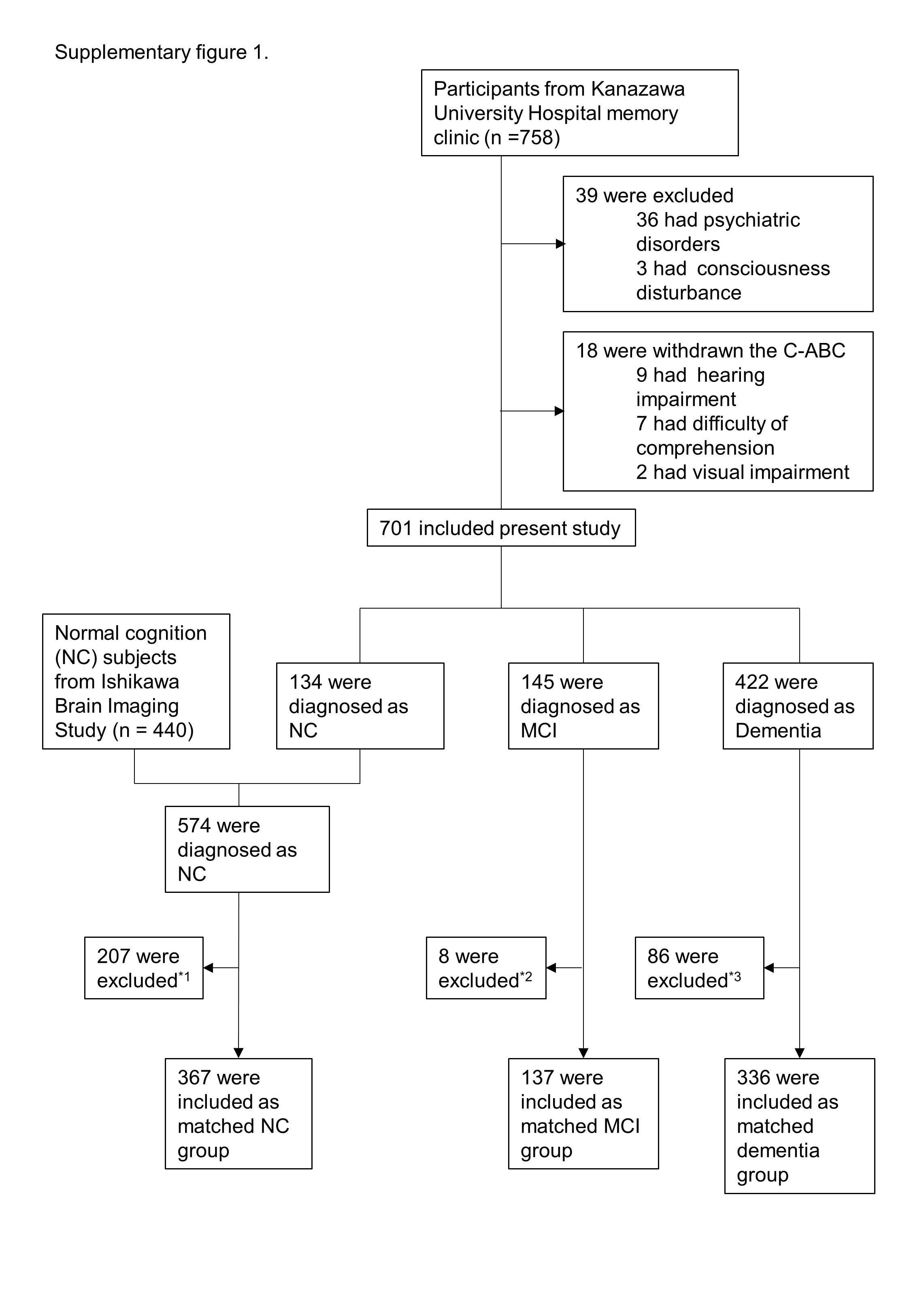

Supplement: S1 Fig — We excluded subjects aged <50 and >85, and analyzed only those 50–85 years, because only 1 subject with MCI aged <50 years and only 2 with NC aged >85 years. We excluded 194 subjects aged <50 years and 2 aged >85 years from the NC group (*1). We excluded 1 subject aged <50 years and 4 aged >85 years from the MCI group (*2). We excluded 6 subjects aged <50 years and 29 aged >85 years from the dementia group (*3). In addition, the dementia, MCI, and NC groups were created that matched age, education period, and gender by random sampling using SPSS software (version 23; SPSS Inc., Chicago, IL). Consequently, we excluded 11, 3, and 51 subjects from the NC (*1), MCI (*2), and dementia (*3) groups, respectively. Overall, we excluded 207 subjects from the NC group, 8 from the MCI group, and 86 from the dementia group, and finally examined 367 subjects as the matched NC group, 137 as the matched MCI group, and 336 as the matched dementia group. C-ABC, computerized assessment battery for cognition; MCI, mild cognitive impairment; NC, normal cognition. (TIF) [file pone.0243469.s001.tif]

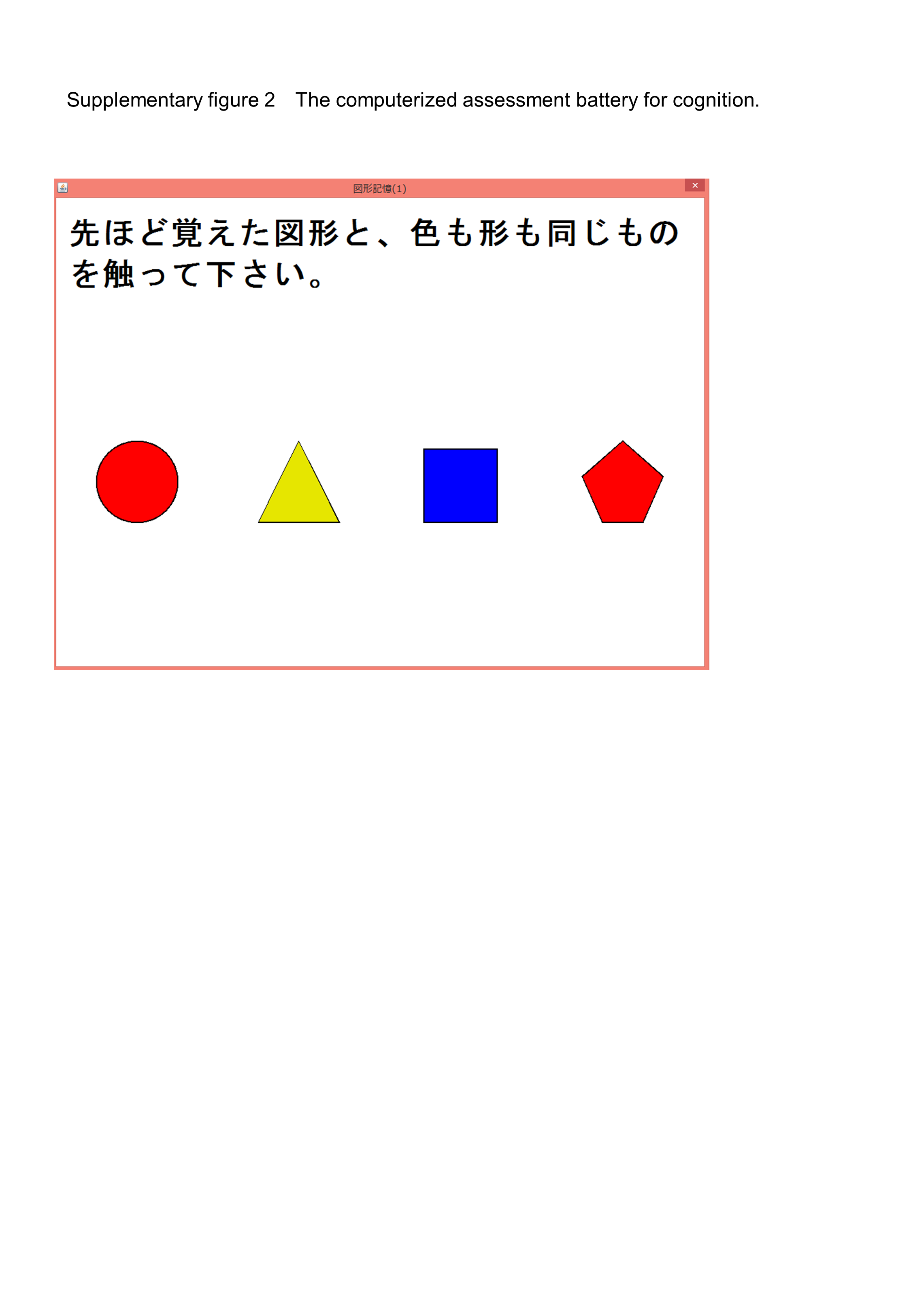

Supplement: S2 Fig — The figures-recognition memory test (item 6). On the PC screen, the question "Please touch the figure with the same color and shape, which you memorized earlier" has been presented. (TIF) [file pone.0243469.s002.tif]

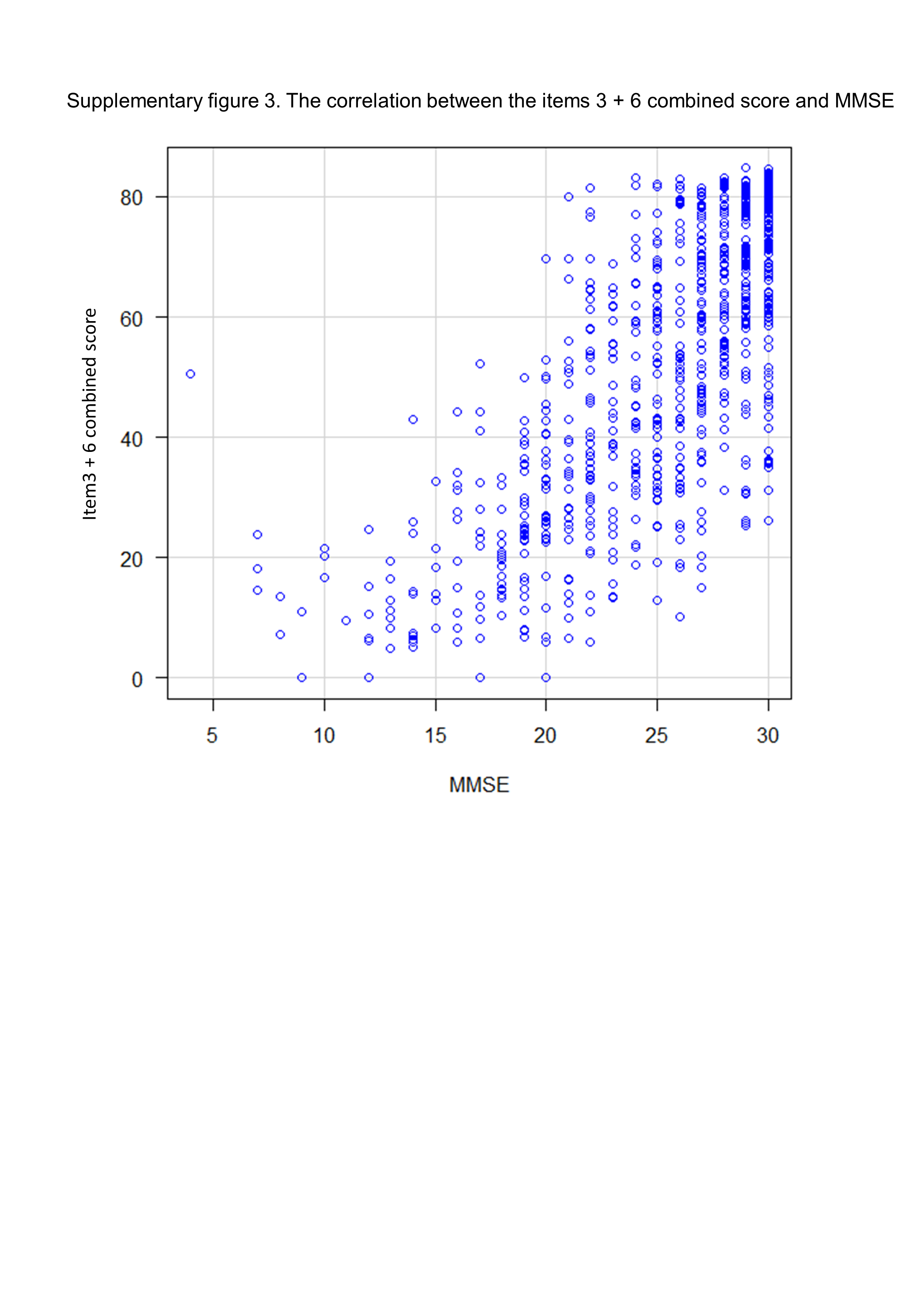

Supplement: S3 Fig — MMSE, Mini-Mental State Examination. (TIF) [file pone.0243469.s003.tif]

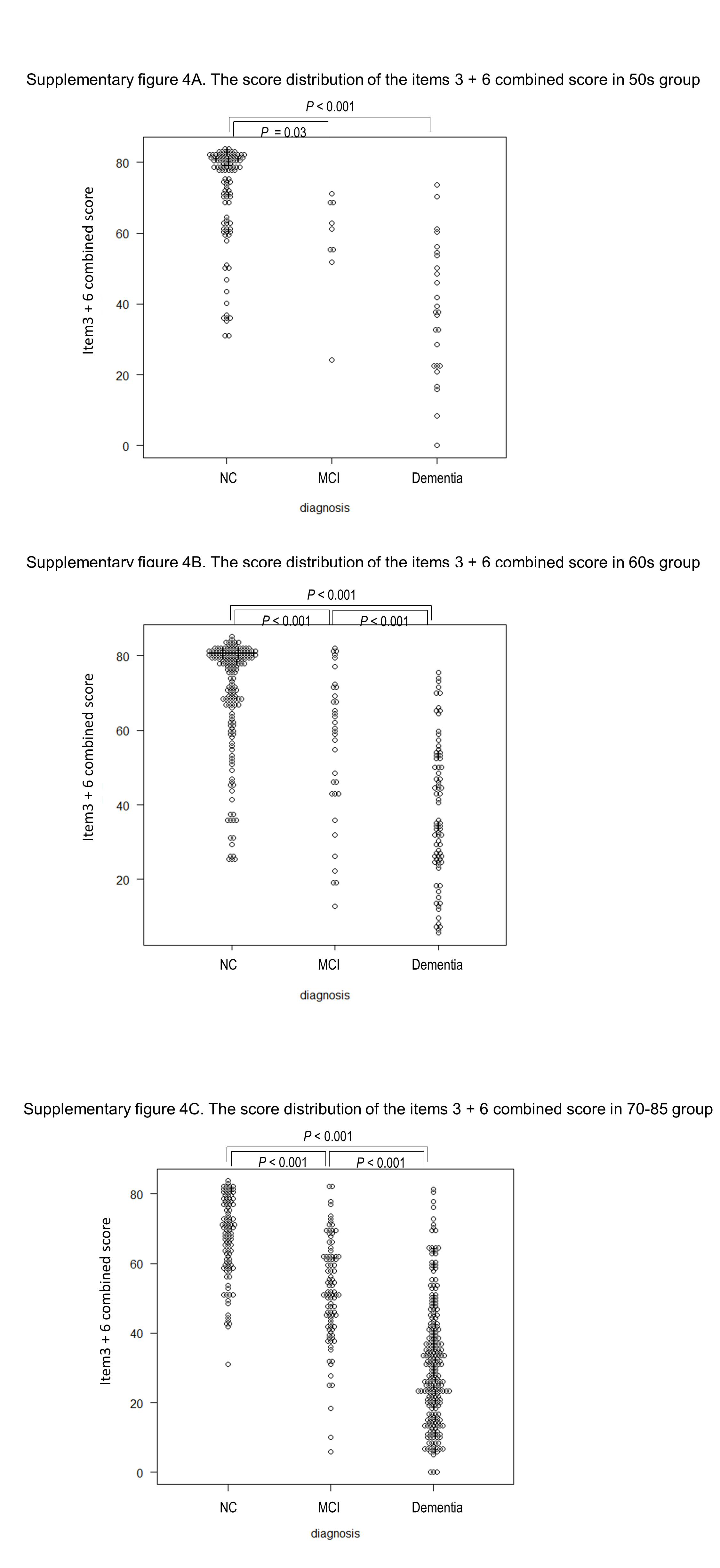

Supplement: S4 Fig — The score distribution of the items 3 + 6 combined score in the 50s group (A), 60s group (B), and 70–85 group (C). The score distribution of C-ABC of NC, MCI, and dementia. C-ABC, computerized assessment battery for cognition; MCI, mild cognitive impairment; NC, normal cognition. (TIF) [file pone.0243469.s004.tif]
