# Supplementary material for: A new computerized assessment battery for cognition (C-ABC) to detect mild cognitive impairment and dementia around 5 min
Source: PLoS One. 2020 Dec 11;15(12):e0243469. doi: 10.1371/journal.pone.0243469 (PMC7732101; doi:10.1371/journal.pone.0243469)
Supplement: S1 Table — (DOCX) [file pone.0243469.s005.docx]

S1 Table. The mean score and percentage of full score, required time, combined score* for each item, all items, and Items 3 + 6 in subjects with NC, MCI, and dementia for the 50s, 60s, and 70–85 groups.

| Mean | NC | MCI | Dementia | *P* value |
| --- | --- | --- | --- | --- |
| 50s group |  |  |  |  |
| Item 1_score (percentage) | 9.98 (99.0) | 10.00 (100.0) | 9.30 (88.5) | 0.012^a^ |
| Item 2_score (percentage) | 9.00 (100.0) | 8.00 (88.9) | 7.96 (88.5) | 0.002^a^ |
| Item 3_score (percentage) | 4.71 (82.4) | 4.44 (77.8) | 3.03 (19.2) | < 0.001^a,b^ |
| Item 4_score (percentage) | 3.78 (85.3) | 3.44 (66.7) | 3.15 (69.2) | 0.005^a^ |
| Item 5_score (percentage) | 2.97 (98.0) | 3.00 (100.0) | 2.84 (92.3) | 0.156 |
| Item 6_score (percentage) | 3.58 (72.5) | 2.88 (44.0) | 2.07 (15.4) | < 0.001^a^ |
| Item 7_score (percentage) | 0.97 (97.1) | 0.88 (88.9) | 0.80 (80.8) | 0.009^a^ |
| Item 8_score (percentage) | 4.00 (100.0) | 4.00 (100.0) | 3.80 (92.3) | 0.042^a^ |
| Total score (percentage) | 39.01 (52.9) | 36.67 (11.1) | 33.00 (0.0) | < 0.001^a^ |
| Item 1_time | 34.57 | 36.96 | 37.48 | 0.058 |
| Item 2_time | 15.37 | 16.40 | 17.27 | 0.032^a^ |
| Item 3_time | 43.52 | 41.54 | 62.29 | < 0.001^a,b^ |
| Item 4_time | 27.54 | 30.46 | 31.23 | 0.059 |
| Item 5_time | 26.54 | 27.02 | 27.04 | 0.059 |
| Item 6_time | 75.96 | 85.47 | 82.63 | < 0.001^a,b^ |
| Item 7_time | 23.85 | 26.82 | 26.25 | < 0.001^a,b^ |
| Item 8_time | 25.50 | 35.12 | 50.52 | 0.001^a^ |
| Total required time | 272.89 | 299.82 | 334.74 | < 0.001^a,c^ |
| Item 1_combined score | 292.95 | 274.53 | 254.71 | 0.001^a^ |
| Item 2_combined score | 602.07 | 499.71 | 486.69 | < 0.001^a^ |
| Item 3_combined score | 117.11 | 105.89 | 59.85 | < 0.001^a,c^ |
| Item 4_combined score | 145.83 | 117.32 | 106.14 | < 0.001^a^ |
| Item 5_combined score | 111.97 | 111.32 | 106.14 | 0.097 |
| Item 6_combined score | 48.18 | 34.00 | 26.11 | < 0.001^a,b^ |
| Item 7_combined score | 41.11 | 33.62 | 32.53 | < 0.001^a^ |
| Item 8_combined score | 170.66 | 131.24 | 119.38 | < 0.001^a,b^ |
| Total combined score | 144.35 | 123.51 | 105.77 | < 0.001^a,b^ |
|  | NC | MCI | Dementia | *P* value |
| Item 3 + 6 combined score | 71.42 | 57.71 | 38.10 | < 0.001^a,b^ |
| 60s group |  |  |  |  |
| Item 1_score (percentage) | 9.95 (99.4) | 9.73 (97.1) | 9.18 (87.0) | 0.001^a^ |
| Item 2_score (percentage) | 8.90 (98.8) | 9.00 (100.0) | 8.51 (89.6) | 0.019^a^ |
| Item 3_score (percentage) | 4.68 (79.4) | 3.94 (50.0) | 2.89 (18.2) | < 0.001^a,b,c^ |
| Item 4_score (percentage) | 3.77 (83.6) | 3.82 (82.4) | 2.81 (48.1) | < 0.001^a,c^ |
| Item 5_score (percentage) | 2.95 (97.6) | 2.76 (91.2) | 2.51 (77.9) | < 0.001^a^ |
| Item 6_score (percentage) | 3.57 (72.7) | 2.91 (41.2) | 3.10 (14.3) | < 0.001^a,b,c^ |
| Item 7_score (percentage) | 0.92 (97.1) | 0.82 (88.9) | 0.48 (80.8) | < 0.001^a,c^ |
| Item 8_score (percentage) | 4.00 (100.0) | 4.00 (100.0) | 3.71 (90.9) | < 0.001^a,c^ |
| Total score (percentage) | 38.77 (50.3) | 37.00 (14.7) | 32.31 (1.3) | < 0.001^a,b,c^ |
| Item 1_time | 35.61 | 36.12 | 38.44 | 0.003^a^ |
| Item 2_time | 15.84 | 16.60 | 20.50 | < 0.001^a,c^ |
| Item 3_time | 45.53 | 52.07 | 60.35 | < 0.001^a^ |
| Item 4_time | 28.90 | 30.47 | 35.13 | < 0.001^a,c^ |
| Item 5_time | 26.73 | 27.97 | 28.44 | < 0.001^a,b^ |
| Item 6_time | 76.65 | 77.42 | 80.11 | 0.018^a^ |
| Item 7_time | 24.49 | 24.84 | 28.32 | < 0.001^a,c^ |
| Item 8_time | 25.78 | 27.30 | 44.73 | < 0.001^a,c^ |
| Total required time | 279.57 | 292.83 | 336.05 | < 0.001^a,b,c^ |
| Item 1_combined score | 284.58 | 270.14 | 239.66 | < 0.001^a,c^ |
| Item 2_combined score | 585.28 | 555.60 | 463.31 | < 0.001^a,c^ |
| Item 3_combined score | 114.73 | 87.24 | 57.05 | < 0.001^a,b,c^ |
| Item 4_combined score | 139.06 | 130.91 | 87.70 | < 0.001^a,c^ |
| Item 5_combined score | 110.78 | 103.06 | 91.90 | < 0.001^a^ |
| Item 6_combined score | 47.46 | 38.20 | 27.68 | < 0.001^a,b,c^ |
| Item 7_combined score | 38.68 | 34.85 | 19.91 | < 0.001^a,c^ |
| Item 8_combined score | 168.46 | 159.24 | 127.78 | < 0.001^a,c^ |
| Total combined score | 140.43 | 127.72 | 100.82 | < 0.001^a,b,c^ |
| Item 3 + 6 combined score | 70.05 | 55.41 | 38.17 | < 0.001^a,b,c^ |
| 70-85 group |  |  |  |  |
| Item 1_score (percentage) | 9.44 (90.0) | 9.36 (86.2) | 8.78 (77.7) | 0.027^a^ |
|  | NC | MCI | Dementia | *P* value |
| Item 2_score (percentage) | 8.72 (96.0) | 8.39 (91.5) | 7.57 (79.8) | < 0.001^a,c^ |
| Item 3_score (percentage) | 4.67 (70.0) | 3.97 (43.6) | 2.35 (9.9) | < 0.001^a,b,c^ |
| Item 4_score (percentage) | 3.68 (74.0) | 3.35 (62.8) | 2.71 (45.1) | < 0.001^a,c^ |
| Item 5_score (percentage) | 2.85 (91.0) | 2.76 (87.2) | 2.42 (72.5) | < 0.001^a,c^ |
| Item 6_score (percentage) | 3.16 (48.0) | 2.41 (14.9) | 1.84 (5.6) | < 0.001^a,b,c^ |
| Item 7_score (percentage) | 0.73 (73.0) | 0.56 (56.4) | 0.46 (46.4) | < 0.001^a^ |
| Item 8_score (percentage) | 3.99 (99.0) | 3.96 (98.9) | 3.61 (87.1) | < 0.001^a,c^ |
| Total score (percentage) | 37.24 (19.0) | 34.80 (4.3) | 29.79 (0.4) | < 0.001^a,b,c^ |
| Item 1_time | 37.16 | 39.82 | 42.77 | < 0.001^a,c^ |
| Item 2_time | 17.27 | 19.13 | 21.95 | < 0.001^a,c^ |
| Item 3_time | 41.99 | 47.02 | 61.41 | < 0.001^a,c^ |
| Item 4_time | 31.80 | 33.24 | 36.26 | < 0.001^a,c^ |
| Item 5_time | 27.59 | 27.88 | 30.23 | < 0.001^a,c^ |
| Item 6_time | 75.34 | 79.37 | 81.82 | < 0.001^a,b^ |
| Item 7_time | 26.56 | 26.92 | 31.76 | < 0.001^a,c^ |
| Item 8_time | 29.06 | 31.16 | 49.81 | < 0.001^a,c^ |
| Total required time | 285.80 | 304.58 | 356.03 | < 0.001^a,b,c^ |
| Item 1_combined score | 258.37 | 242.54 | 212.72 | < 0.001^a,c^ |
| Item 2_combined score | 528.78 | 475.79 | 385.20 | < 0.001^a,c^ |
| Item 3_combined score | 113.28 | 90.59 | 47.10 | < 0.001^a,b,c^ |
| Item 4_combined score | 124.76 | 108.55 | 81.90 | < 0.001^a,c^ |
| Item 5_combined score | 105.12 | 101.99 | 86.66 | < 0.001^a,c^ |
| Item 6_combined score | 42.29 | 30.99 | 23.00 | < 0.001^a,b,c^ |
| Item 7_combined score | 30.09 | 22.96 | 17.25 | < 0.001^a,b,c^ |
| Item 8_combined score | 155.86 | 148.16 | 117.12 | < 0.001^a,c^ |
| Total combined score | 131.74 | 116.23 | 88.81 | < 0.001^a,b,c^ |
| Item 3 + 6 combined score | 67.23 | 51.86 | 31.16 | < 0.001^a,b,c^ |

^a^*P* < 0.05, NC vs. dementia.

^b^*P* < 0.05, NC vs. MCI.

^c^*P* < 0.05, MCI vs. dementia.

Brackets means percentage of the full score.

*C-ABC total and each item combined scores were calculated by dividing the C-ABC total and each item score by the total and each item required time (s) and multiplying by 1000, respectively.
