# Supplementary material for: A new computerized assessment battery for cognition (C-ABC) to detect mild cognitive impairment and dementia around 5 min
Source: PLoS One. 2020 Dec 11;15(12):e0243469. doi: 10.1371/journal.pone.0243469 (PMC7732101; doi:10.1371/journal.pone.0243469)
Supplement: S2 Table — (DOCX) [file pone.0243469.s006.docx]

S2 Table. Multiple logit estimates for dementia or MCI

|  | Dementia from MCI and NC | | | |  | MCI from NC | | | |
| --- | --- | --- | --- | --- | --- | --- | --- | --- | --- |
|  | Coefficient | SE | Standardized coefficient | P value |  | Coefficient | SE | Standardized coefficient | P value |
| Age | 0.000 | 0.002 | 0.006 | 0.829 |  | 0.010 | 0.002 | 0.187 | < 0.001 |
| Sex | -0.018 | 0.025 | -0.018 | 0.472 |  | -0.059 | 0.034 | -0.066 | 0.089 |
| Education period | 0.001 | 0.001 | 0.028 | 0.262 |  | 0.000 | 0.002 | -0.007 | 0.860 |
| Item 1_combined score | 0.000 | 0.000 | -0.048 | 0.110 |  | 0.000 | 0.000 | -0.059 | 0.172 |
| Item 2_combined score | 0.000 | 0.000 | -0.056 | 0.083 |  | 0.000 | 0.000 | -0.069 | 0.100 |
| Item 3_combined score | -0.005 | 0.000 | -0.433 | < 0.001 |  | -0.003 | 0.001 | -0.212 | < 0.001 |
| Item 4_combined score | 0.000 | 0.000 | -0.039 | 0.235 |  | 0.000 | 0.000 | -0.011 | 0.796 |
| Item 5_combined score | 6.423E-5 | 0.000 | 0.004 | 0.897 |  | 0.000 | 0.001 | -0.006 | 0.882 |
| Item 6_combined score | -0.006 | 0.001 | -0.197 | < 0.001 |  | -0.007 | 0.001 | -0.229 | < 0.001 |
| Item 7_combined score | -0.002 | 0.001 | -0.066 | 0.024 |  | -0.002 | 0.001 | -0.062 | 0.150 |
| Item 8_combined score | -0.001 | 0.000 | -0.058 | 0.052 |  | 0.000 | 0.000 | -0.030 | 0.456 |

MCI: mild cognitive impairment; NC: normal cognition
