# Supplementary material for: A new computerized assessment battery for cognition (C-ABC) to detect mild cognitive impairment and dementia around 5 min
Source: PLoS One. 2020 Dec 11;15(12):e0243469. doi: 10.1371/journal.pone.0243469 (PMC7732101; doi:10.1371/journal.pone.0243469)
Supplement: S3 Table — (DOCX) [file pone.0243469.s007.docx]

S3 Table. Measure of the diagnostic accuracy of items 3 + 6 combined score to distinguish MCI from NC and dementia from MCI and NC by ROC analyses.

|  | MCI from NC | | |  | Dementia from MCI and NC | | |
| --- | --- | --- | --- | --- | --- | --- | --- |
| items 3 + 6 combined score | Specificity ≥ 0.9 | OCV | Sensitivity ≥ 0.9 |  | Specificity ≥ 0.9 | OCV | Sensitivity ≥ 0.9 |
| 50s group |  |  |  |  |  |  |  |
| Cutoff point | 46.69 | 71.42 | 80.86 |  | 48.82 | 60.92 | 68.23 |
| Sensitivity | 0.11 | 1.00 | 1.00 |  | 0.69 | 0.92 | 0.92 |
| Specificity | 0.90 | 0.66 | 0.26 |  | 0.90 | 0.78 | 0.69 |
| 60s group |  |  |  |  |  |  |  |
| Cutoff point | 44.10 | 67.56 | 80.07 |  | 36.17 | 59.34 | 65.62 |
| Sensitivity | 0.29 | 0.70 | 0.91 |  | 0.53 | 0.87 | 0.90 |
| Specificity | 0.90 | 0.71 | 0.32 |  | 0.90 | 0.76 | 0.68 |
| 70–85 group |  |  |  |  |  |  |  |
| Cutoff point | 43.78 | 62.11 | 69.74 |  | 36.04 | 40.91 | 57.85 |
| Sensitivity | 0.27 | 0.78 | 0.90 |  | 0.66 | 0.75 | 0.90 |
| Specificity | 0.95 | 0.69 | 0.48 |  | 0.93 | 0.89 | 0.60 |

MCI, mild cognitive impairment; NC, normal cognition; OCV, optimal cutoff value (in this study, OCV implies the value in which the sum of sensitivity and specificity reaches the maximum); ROC, receiver-operating characteristic analysis
